# A role for the dehydrogenase DHRS7 (SDR34C1) in prostate cancer

**DOI:** 10.1002/cam4.517

**Published:** 2015-08-26

**Authors:** Julia K Seibert, Luca Quagliata, Cristina Quintavalle, Thomas G Hammond, Luigi Terracciano, Alex Odermatt

**Affiliations:** 1 Division of Molecular and Systems Toxicology, Department of Pharmaceutical Sciences, University of BaselKlingelbergstrasse 50, CH-4056, Basel, Switzerland; 2 Molecular Pathology Division, Institute of Pathology, University Hospital and University of BaselSchönbeinstrasse 40, CH-4003, Basel, Switzerland

**Keywords:** DHRS7, gene expression, proliferation, prostate cancer, SDR34C1, tumor suppressor

## Abstract

Several microarray studies of prostate cancer (PCa) samples have suggested altered expression of the “orphan” enzyme short-chain dehydrogenase/reductase *DHRS7* (retSDR4, SDR34C1). However, the role of DHRS7 in PCa is largely unknown and the impact of DHRS7 modulation on cancer cell properties has not yet been studied. Here, we investigated DHRS7 expression in normal human prostate and PCa tissue samples at different tumor grade using tissue microarray and immunovisualization. Moreover, we characterized the effects of siRNA-mediated DHRS7 knockdown on the properties of three distinct human prostate cell lines. We found that DHRS7 protein expression decreases alongside tumor grade, as judged by the Gleason level, in PCa tissue samples. The siRNA-mediated knockdown of DHRS7 expression in the human PCa cell lines LNCaP, BPH1, and PC3 significantly increased cell proliferation in LNCaP cells as well as cell migration in all of the investigated cell lines. Furthermore, cell adhesion was decreased upon DHRS7 knockdown in all three cell lines. To begin to understand the mechanisms underlying the effects of DHRS7 depletion, we performed a microarray study with samples from LNCaP cells treated with *DHRS7*-specific siRNA. Several genes involved in cell proliferation and adhesion pathways were found to be altered in DHRS7-depleted LNCaP cells. Additionally, genes of the BRCA1/2 pathway and the epithelial to mesenchymal transition regulator E-cadherin were altered following DHRS7 knockdown. Based on these results, further research is needed to evaluate the potential role of DHRS7 as a tumor suppressor and whether its loss-of-function promotes PCa progression and metastasis.

## Introduction

Prostate cancer (PCa) is one of the most common malignancies worldwide, with its incidence continually rising. It is also the second leading cause of cancer-related death among men 1. Clinically, PCa manifests as a heterogeneous, multifocal disease 2,3. The mechanisms underlying the initiation and progression of PCa are complex. Initially, premalignant lesions, which are attributed to genetic alterations in one or more cells, arise. Subsequently, genetic alterations can occur in one or a few of the premalignant cells, resulting in changes of signaling pathways and resulting in malignant growth and the formation of a primary tumor. Cells in a primary tumor are heterogeneous regarding their phenotypic and biological characteristics, caused by the differences in the genes that were affected, thus making therapeutic interventions challenging.

Besides environmental factors, age and familial inherited susceptibility factors, steroid hormone receptor signaling plays a pivotal role in all stages of prostate carcinogenesis. The androgen receptor (AR) signaling pathway is thought to promote the early development of PCa, and it also has an important role in the development of castration resistant prostate cancer (CRPC), which fails to respond to hormone deprivation therapies. Several mechanisms have been suggested to cause the progression of PCa to CRPC, including hypersensitivity of the AR signaling pathway to androgens, enrichment or accumulation of androgen-insensitive stem cells, and activation of intratumoral steroidogenesis 4. Furthermore, there is evidence for a pivotal role of the process of epithelial–mesenchymal transition (EMT) in the development of metastatic CRPC 5.

In its initial stages, PCa is often curable; however, despite recent advances in current therapeutic methods, many patients develop postoperative disease relapse and suffer from significant treatment-associated complications. The current standard for the treatment of PCa is either medical or surgical castration. Nevertheless, following castration, PCa can progress to CRPC and patients may develop metastases in various organs such as lymph nodes, liver, and bone 6,7. Besides the androgen-dependent growth stimulation, a comprehensive understanding of the molecular mechanisms underlying PCa progression is largely incomplete, with further research warranted. Thus, it is important to investigate new players in PCa to understand disease progression and develop improved strategies for the prevention and therapy.

In this context, analyses of the transcriptome of PCa samples can provide hints to genes involved in cancer development and progression. Microarray expression data from three independent studies on human PCa tissues suggested that the levels of the short-chain dehydrogenase/reductase (SDR) enzyme DHRS7 (also known as retSDR4 and under the nomenclature name SDR34C1) 8 are frequently altered in this tumor 9–11. A recent study of the transcriptome of human LNCaP PCa cells hypothesized that DHRS7, among several other genes, may play a role in sustaining de novo androgen synthesis and/or metabolism in CRPC, eventually leading to the reactivation of AR, thus promoting cancer progression even upon ablation of testicular androgen production 12.

DHRS7 was initially cloned from retinal pigment epithelium cells 13; however, it is expressed in various tissues including the prostate 14,15. Little is known on the catalytic activity and physiological role of DHRS7 and this enzyme has therefore to be considered as an “orphan” SDR. A recent study by Stambergova et al. suggested that DHRS7 possesses NADP(H) cofactor preference and enzymatic reducing activity toward endogenous substrates with a steroid structure (estrone, cortisone, and 4-androstene-3,17-dione) and exogenous substances bearing a carbonyl group (1,2-naphtoquinone, 9,10-phenanthrenequinone, benzoquinone, and nitrosamine 4-(methyl-nitrosamino)-1-(3-pyridyl)-1-butanone) 16. However, the evidence for a role of DHRS7 in the metabolism of these compounds was based on indirect measurements of NADPH consumption. Other investigators did not observe any activity toward steroids and retinoids 13. Nevertheless, some of the closest relatives of DHRS7, namely 11*β*-HSD and 17*β*-HSD enzymes, have been associated with cancer. Particularly, 17*β*-HSD1, 17*β*-HSD2, and 17*β*-HSD12, with ∼25–40% sequence homology, are involved in the control of the ratio of active to inactive estrogens and androgens, and they are known to play a role in prostate and breast cancer, letting us to hypothesize that DHRS7 function might affect tumor growth 17–19.

Based on the preliminary observations from microarray studies and the relation of DHRS7 to SDRs involved in PCa, we investigated the role of DHRS7 in PCa, taking advantage of a combination of human data obtained on a large cohort (*n* = 348) of samples on tissue microarrays [20,21] and in vitro experiments comprising modulation of DHRS7 expression by knockdown. To determine the effects of DHRS7 on the aggressiveness of prostate cells in vitro we evaluated cell proliferation, migration, and adhesion after siRNA-mediated knockdown in three prostate cell lines, namely LNCaP, BPH1, and PC3. Furthermore, we performed a microarray experiment using LNCaP cells treated with siRNA against DHRS7 in order to obtain initial insights into the pathways involved in its action.

## Material and Methods

### TMA construction and clinical pathology data

The use of clinical specimens for the construction of tissue microarrays (TMAs) was approved by the ethical committee of the University Hospital of Basel, Switzerland. The TMAs were manufactured as described previously 22,23. Briefly, the PCa progression TMAs consist of formalin-fixed and paraffin-embedded specimens obtained from 551 PCa patients who were treated for clinically localized PCa by radical prostatectomy or transurethral resection (TURP) plus 68 normal prostate tissues. One core tissue-biopsy per each of the 551 patients’ blocks (diameter 0.6 mm) was taken from the least differentiated region of individual paraffin-embedded prostate tumors (donor blocks) and arrayed into a new recipient paraffin block (35 and 20 mm). Because of their small size, Gleason grade rather than Gleason score was assigned to the specimens on the TMA sections. To evaluate DHRS7 protein expression the TMAs were stained with an anti-DHRS7 antibody (rabbit anti-human DHRS7 polyclonal antibody, HPA031121; Sigma, St. Louis, MO, 1:200 dilution) and analyzed by an experienced pathologist (L. T.). Reference for protein staining optimization and controls can be found at: http://www.proteinatlas.org/ENSG00000100612-DHRS7/tissue. Immunoreactivity was scored semi-quantitatively (0 = negative and 3 = highest intensity) by evaluating the staining intensity as described by Allred et al. 24.

### Cell lines and cell culture

The human prostate carcinoma cell line LNCaP was newly purchased from ATCC (LGC Standards GmbH, Wesel, Germany). PC3 cells and the benign prostate hyperplastic cell line BPH1 were available in-house and originally purchased from ATCC. The identity of the cell lines was verified by the multiplex human cell line authentication test (Multiplexion, Immenstaad, Germany). All cell lines were maintained in RPMI 1640 (R8758; Sigma) supplemented with 10% fetal bovine serum (FBS) and penicillin (100 U/mL)/streptomycin (100 *μ*g/mL). Cells were cultured at 37°C in a 5% CO_2_ atmosphere.

### Immunohistochemical staining

LNCaP, PC3, and BPH1 cells were seeded in six-well plates containing a 18-mm round glass slide (Menzal-Glaser, Braunschweig, Germany) at 3 × 10^5^ cells per well. For indirect immunofluorescence experiments, culture medium was removed and cells were washed twice with phosphate buffered saline (PBS), fixed with 4% paraformaldehyde for 15 min and washed three times with PBS. Cells were permeabilized with 0.1% Triton X-100 for 10 min and blocked with 1% bovine serum albumin for 20 min at room temperature. Blocking was followed by incubation with a primary antibody against DHRS7 (rabbit anti-human DHRS7 polyclonal antibody; 1:500 dilution in 1% bovine serum albumin, HPA031121; Sigma) for 1 h at room temperature. After three washes with PBS, Hoechst-33342 (5 *μ*g/mL, H3570; Invitrogen Life Technologies, Zug, Switzerland) and goat anti-rabbit HiLyte™ Fluor 488-labeled (1:2000, AS-61056-1-H488; Anaspec, Fremont, CA) secondary antibody was applied for 30 min at room temperature. Cells were washed three times with PBS, mounted in Mowiol 4-88, and slides were analyzed under a laser scanning confocal microscope (Fluoview 1000; Olympus, Shinjuku, Japan).

### Transfection with siRNA

LNCaP, PC3, and BPH1 cells (3 × 10^5^) were reverse transfected on a six-well plate using Lipofectamine RNAiMax reagent with 10 nmol/L of siRNA targeting *DHRS7* (D-009573-02; Thermo Scientific, Waltham, MA) or a nontargeting siRNA negative control (D-001810-03-20; Thermo Scientific). Effective knockdown was verified by qPCR as well as western blot and immunodetection. To choose the siRNA for the main experiments, we performed preliminary knockdown experiments with four different siRNAs and a pool of all of them (MQ-009573-00; Thermo Scientific), and determined the most effective knockdown by qPCR after 24, 48, and 72 h (Fig. S1). To ensure that the siRNA effects observed for the specific siRNA (D-009573-02) were due to DHRS7 knockdown and not off-target effects, we additionally performed the proliferation assay in LNCaP with another DHRS7-specific siRNA (D-009573-04) (Fig. S2). The results obtained were similar for both tested siRNAs. For the functional assays, cells were used at 24 h posttransfection.

### Real-time qPCR

Total RNA was isolated from cultured cells using TRI-reagent (T9424; Sigma) according to the manufacturer’s instructions. RNA quality and quantity was measured using a NanoDrop ND-1000 spectrometer (NanoDrop Technologies, Wilmington, DE). Reverse transcription was performed using the M-MLV Reverse Transcriptase Kit (M368B; Promega, Wallisellen, Switzerland) according to the manufacturer’s instructions. Relative quantification of mRNA expression levels was performed by real-time qPCR. Briefly, cDNA (10 ng), gene-specific oligonucleotide primers (Table S1) (200 nmol/L), and KAPA SYBR FAST qPCR reagent (KK4600; Kapa systems, Wilmington, DE) (5 *μ*L), in a final volume of 10 *μ*L, were analyzed by qPCR in a rotor gene 3000A (Corbett Research, Sydney, Australia). Thermal cycler parameters were as follows: denaturation for 15 min at 95°C, followed by amplification of cDNA for 40 cycles with melting for 15 sec at 94°C, annealing for 30 sec at 56°C, and extension for 30 sec at 72°C. Relative gene expression normalized to the internal control gene coding for cyclophilin A (PPIA) was obtained by the 2^−ΔΔCt^ method 23.

### Western blot

Cells were lysed using RIPA buffer (R0278; Sigma) and centrifuged at 12,000*g* for 10 min at 4°C. The supernatant was collected and protein concentration quantified using the Pierce® biocinchonic acid protein assay kit (23225; Thermo Scientific). Samples were heated at 95°C for 5 min in Laemmli buffer (5 mmol/L Tris HCl, pH 6.8, 10% glycerol [v/v], 0.2% sodium dodecyl sulfate [SDS] [w/v], 1% bromophenol blue [w/v]) and stored at −20°C until used. Lysates were separated by a 12.5% Tris-glycine SDS-polyacrylamide gel, and transferred to Immun-Blot® polyvinylidene difluoride membranes (162-0177; Bio-Rad Laboratories, Hercules, CA) at constant 230 mA for 1 h. For detection of DHRS7, the membrane was blocked using 2% milk (v/v) for 1 h at room temperature, followed by incubation with the mouse anti-human DHRS7 polyclonal antibody (ab69348; Abcam, Cambridge, UK) at a dilution of 1:500 (v/v) in 2% milk (v/v), overnight at 4°C. After washing with Tris-buffered saline (20 mmol/L Tris-base, 140 mmol/L NaCl) containing 0.1% Tween-20 (v/v) (TBS-T), the membrane was subsequently incubated with horseradish peroxidase-conjugated goat anti-mouse secondary antibody (Jackson Immuno Research, Suffolk, UK) for 1 h at room temperature. For PPIA detection, the membrane was blocked using 10% milk (v/v) overnight at 4°C, followed by incubation with the rabbit anti-human PPIA polyclonal antibody (ab41684; Abcam) at a dilution of 1:2000 (v/v) in 2% milk for 1 h at room temperature. After washing with TBS-T, the membrane was subsequently incubated with horseradish peroxidase-conjugated goat anti-rabbit secondary antibody (Santa Cruz Biotechnology, Santa Cruz, CA) at a dilution of 1:1000 (v/v) in 2% milk (v/v). After washing the membranes in TBS-T, images were visualized using the Immobilon Western Chemiluminescent HRP substrate kit (Millipore, Schaffhausen, Switzerland), and a FujiFilm ImageQuant™ LAS-4000 detector (GE Healthcare, Glattbrugg, Switzerland) using the chemiluminescence detection setting.

### xCELLigence cell proliferation assay

The xCELLigence DP device (ACEA Biosciences, San Diego, CA) was used to monitor cell proliferation in real-time. LNCaP, PC3, and BPH1 cells were seeded in E-plates (E-Plate View™; ACEA) at 1  ×  10^4^, 5 ×  10^3^, and 5 × 10^3^ cells per well, respectively. Proliferation was determined kinetically over 48 h using the xCELLigence system according to the manufacturer’s protocol. Cell proliferation measurements were performed in triplicates with programmed signal detection every 15 min. Data acquisition and analyses were performed using the RTCA software (version 1.2; ACEA).

### Ki-67 cell proliferation assay

Following 24 h after DHRS7 siRNA transfection, cells were detached, diluted to 10,000 cells in 100 *μ*L, and placed onto a Superfrost™ microscope slide using a Cytospin™ centrifuge (Thermo Scientific). The slides were fixed in delaune for 2 min and then left to dry at room temperature for 5 min. Ki-67 staining was performed with a BenchMark Ultra platform automated immunohistochemistry/in situ hybridization (IHC/ISH) staining system (Roche, Rotkreuz, Switzerland). Ki-67 index was determined by ascertaining the percentage of Ki-67 positively stained cells in five fields scanned at 20× magnifications using ImageJ software (http://imagej.nih.gov/ij/).

### Transwell migration assay

LNCaP, PC3, and BPH1 cells were reseeded 24 h posttransfection into the top chamber of a 24-well noncoated insert (pore size 8 *μ*m; Corning Inc., Lowell, MA) at 1 × 10^5^, 0.5 × 10^5^, and 1 × 10^5^ cells per well, respectively. The upper chamber contained RPMI media supplemented with 1% FBS, whereas the bottom chamber contained 10% FBS as a chemoattractant. Following 24 h of incubation, cells were stained with 0.1% crystal violet (w/v) (C3886; Sigma) in 25% methanol (v/v). Nonmigrated cells in the upper chamber were removed using a cotton swab. Images of migrated cells which adhered to the bottom of the filter were captured at 10× magnification using a light microscope (Zeiss Axiovert 100; Carl Zeiss Microscopy GmbH, Feldbach, Switzerland) and relative areas of staining were assessed using the threshold setting on Image J.

### Cell adhesion

Ninety-six-well plates were coated with 50 *μ*g/mL fibronectin and blocked with 0.5% bovine serum albumin in RPMI medium for 45 min at room temperature. LNCaP, PC3, and BPH1 cells were seeded at 1 × 10^5^ cells per well and allowed to adhere for 60 min. Wells were then washed twice with PBS. The number of adherent cells in each well was quantified through staining with 0.1% crystal violet (w/v) in 25% methanol (v/v), followed by optical density (OD) measurement.

### cRNA target synthesis and gene chip hybridization

Total RNA for the microarray was isolated with Direct-Zol RNA MiniPrep Kit (Zymo Research, Irvine, CA) including on-column DNAse treatment. RNA concentration was assessed using a NanoDrop ND 1000 (NanoDrop) and RNA integrity was monitored on a Bioanalyzer RNA 6000 Chip (Agilent, Basel, Switzerland). DNAse-treated total RNA (270 ng) was subjected to target synthesis using the WT Expression kit (Life Technologies), following the manufacturer’s recommendations. Fragmentation and labeling of amplified cDNA was performed using the WT Terminal Labeling Kit (Affymetrix, Santa Clara, CA). Synthesis reactions were carried out using a PCR machine (TProfessionnalTrio; Biometra, Goettingen, Germany) in 0.2 mL tubes. Eighty-five microliters of cocktail containing 23.4 ng/*μ*L labeled DNA was loaded on a Affimetrix GeneChip Human Gene 2.0 ST Array (Cat# 902499) and hybridized for 17 h (45°C, 60 rpm) in a hybridization oven 645 (Affymetrix). These gene chips have the particularity of interrogating all well-established annotation RefSeq coding transcripts (26,191) and in addition many well-established annotation RefSeq noncoding transcripts (3391). The arrays were washed and stained on a Fluidics Stations 450 (Affymetrix), using the Hybridization Wash and Stain Kit according to the FS450_0002 protocol (Affymetrix). The gene chips were scanned with an Affymetrix GeneChip Scanner 3000 7G. DAT images and CEL files of the microarrays were generated using Affymetrix GeneChip Command Control software (version 4.0). Afterward, CEL files were imported into Qlucore software (Qlucore AB, Lund, Sweden) and robust multichip average normalized. Subsequently, principal component analysis to discriminate between engineered and control cells was performed. Quantile normalization and data processing were performed using the GeneSpringGXv11.5.1 software package (Agilent). The gene signature value was assessed using the BRB-ArrayTool (v4.3.2; NIH, Bethesda, MD). Ingenuity software (Qiagen, Venlo, the Netherlands) was use to perform pathways analysis.

### Statistics

For the statistical analysis, the chi-square test and the Fisher’s exact test for nonparametric variables and analysis of variance (ANOVA) or Student’s *t*-test for parametric variables were used, with all probabilities reported as two-tailed. Differences in patient survival were assessed using the Kaplan–Meier method and analyzed using the log-rank test in univariate analysis. All tests were two sided and *P* < 0.05 considered being statistically significant. Cutoff scores were selected by evaluating the receiver-operating characteristic curves. The point on the curve with the shortest distance to the coordinate (0, 1) was selected as the threshold value to classify cases as “positive/overexpressing” or “negative/downregulated”. Analyses were performed using the SPSS software (IBM, Armonk, NY).

## Results

### DHRS7 expression is downregulated in human PCa with increasing tumor grade

**Figure 1:**
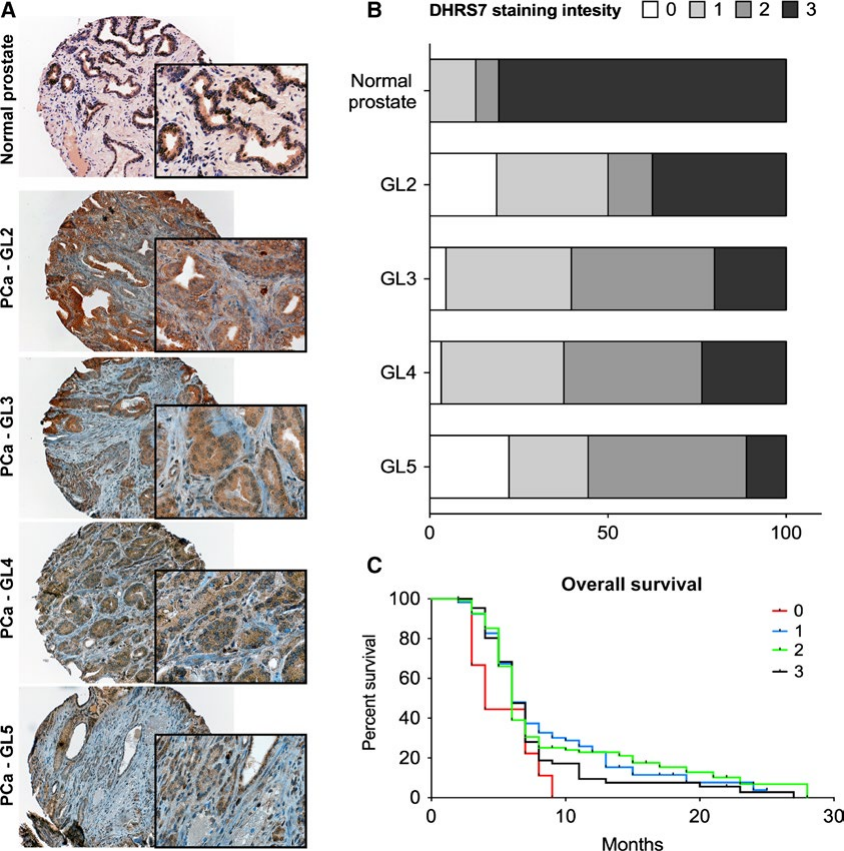
DHRS7 expression in human prostate samples. (A) Representative pictures (taken at 20× and magnified at 60× in the boxes) of DHRS7 staining intensity in normal prostate versus prostate cancer (PCa) with different Gleason levels (GL2 to GL5). Formalin-fixed and paraffin-embedded prostate specimens were analyzed using a rabbit polyclonal anti-human DHRS7 antibody. (B) DHRS7 staining intensity distribution plots highlight that DHRS7 expression is reduced in PCa compared with normal prostate. (C) Kaplan–Meier curves based on DHRS7 expression levels suggest no major impact on the survival of PCa patients.

To evaluate a potential role of DHRS7 in PCa, DHRS7 protein levels were analyzed by immunohistochemistry with a rabbit polyclonal anti-human DHRS7 antibody in a large collection of human prostate specimens using a set of TMAs. Representative pictures of DHRS7 staining are shown in Figure1A and Figure S3. Of the 491 stained tissue punches of PCa, 326 were suitable for analysis and 31 of the 68 tissue punches from normal prostate could be used for this study. Tissues were excluded either as a consequence of poor staining quality or loss of the section from the slide. These analyses revealed DHRS7 to be highly expressed in normal prostate, with the vast majority of analyzed samples (80.6%) classified as benign with intensity three (scoring system: 0 = negative for DHRS7 and 3 = highest intensity of DHRS7, as described under Material and Methods section). Conversely, most of the PCa specimens were scored having either score 2 or 1, 39% and 34.4%, respectively (Fig.1B). Complete loss of DHRS7 was never observed in normal prostate tissue samples, while this was the case in 6.1% of PCa samples. Further stratification of PCa samples, based on their Gleason level (GL), outlined that the group of patients with the highest GL, namely GL5, presented with the lowest percentage of score 3 DHRS7 specimens (22.2%) (Table1). These results suggest that the loss of DHRS7 is associated with PCa and tumor grade. Nevertheless, Kaplan–Meier plots did not reveal a significant association between DHRS7 expression and the survival of PCa patients (Fig.1C).

**Table 1:** Summary of observed DHRS7 protein levels in normal prostate and PCa samples

DHRS7 intensity *n*						
Tissue type	0	1	2	3	*χ* ^2^	*P*
Normal prostate	0	4	2	25		
PCa–GL2	3	5	2	6	11.10	<0.01
PCa–GL3	9	70	80	40	49.90	<0.0001
PCa–GL4	3	32	36	22	32.52	<0.0001
PCa–GL5	4	4	8	2	25.54	<0.0001

Stratification of PCa samples based on their GL was performed by semiquantitatively evaluating the immunostaining intensity as described by Allred et al. 22. Normal prostate (*n* = 31), PCa–GL2 (*n* = 16), PCa–GL3 (*n* = 199), PCa–GL4 (*n* = 93), and PCa–GL5 (*n* = 18). Results were analyzed using chi-square test and *P*-values were calculated referring each PCa group to the normal prostate control group. PCa, prostate cancer; GL, Gleason level.

### Impact of DHRS7 knockdown on the proliferation of PCa cells

**Figure 2:**
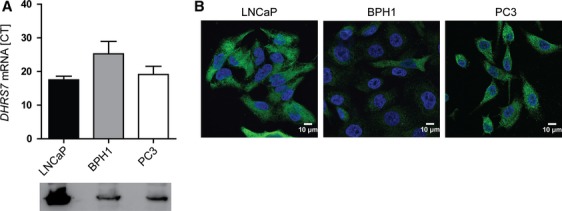
Endogenous expression of DHRS7 in LNCaP, BPH1, and PC3 cells as assessed by qPCR, western blot, and immunovisualization. (A) For qPCR, 10 ng cDNA was used and data were normalized to PPIA control. For western blotting, an amount of 40 *μ*g of total protein was separated by SDS-PAGE, proteins were transferred onto PVDF membranes, followed by detection using a mouse polyclonal anti-human DHRS7 antibody. (B) Immunofluorescence staining was performed using a rabbit polyclonal anti-human DHRS7 antibody. Nuclei were stained with Hoechst-33342. SDS-PAGE, sodium dodecyl sulfate polyacrylamide gel electrophoresis; PVDF, polyvinylidene difluoride.

Since DHRS7 expression decreases significantly as the tumor grade increases, we investigated the functional effects of silencing DHRS7 expression in prostate cell lines endogenously expressing DHRS7 at different amounts, as determined by real-time PCR, western blotting and immunovisualization, namely LNCaP (high), PC3 (moderate), and BPH1 (low) (Fig.2).

**Figure 3:**
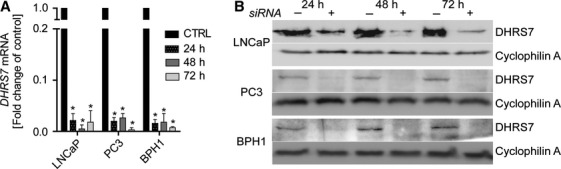
Knockdown of DHRS7 in prostate cell lines. Real-time qPCR (A) and western blot (B) showed the efficacy of siRNA against DHRS7 compared with that of control siRNA after 24, 48, and 72 h in LNCaP, BPH1, and PC3 cells. (A) For real-time PCR, 10 ng cDNA was used. PPIA served as a house-keeping control. Results are expressed as fold change of control and represent mean ± SD from three independent experiments conducted in triplicates. Statistical analysis was determined using the Student’s *t*-test. *P ≤ 0.0001. (B) For western blot, 10 *μ*g of total protein for LNCaP and 30 *μ*g for BPH1 and 37 *μ*g for PC3 were subjected to sodium dodecyl sulfate polyacrylamide gel electrophoresis and western blotting using a mouse polyclonal anti-human DHRS7 antibody. Representative experiments are shown.

SiRNA-mediated targeting efficiently depleted *DHRS7* mRNA expression by more than 90% at all investigated time points (Fig.3A). The impact of siRNA-mediated knockdown of *DHRS7* gene expression on protein levels was assessed by western blotting (Fig.3B). Although the mouse anti-human DHRS7 polyclonal antibody used showed limited sensitivity in western blots, it was preferable over the rabbit anti-human DHRS7 polyclonal antibody and allowed qualitative assessment of protein expression. Protein levels were reduced to ∼30–50% after 24 h and to below 10–20% after 48 and 72 h in siRNA treated LNCaP cells. Knockdown of DHRS7 protein expression was also demonstrated in PC3 and BHP1 cells, whereby a very faint band was still detectable after 24 h but no longer after 48 and 72 h. No morphological changes were observed following depletion of DHRS7 in these prostate cell lines (data not shown).

**Figure 4:**
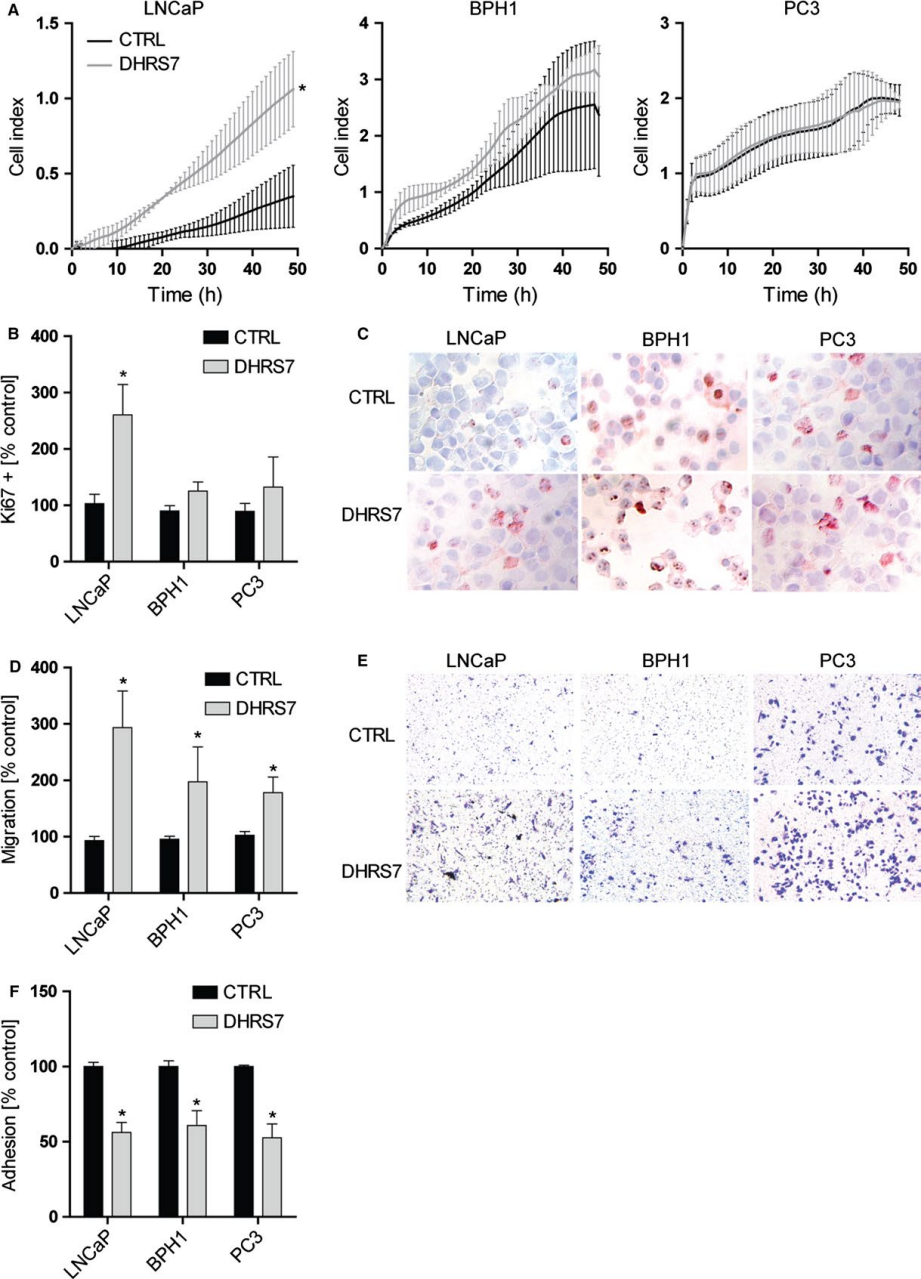
Influence of DHRS7 knockdown on proliferation, migration, and adhesion in LNCaP, BPH1, and PC3 cells. (A) The xCELLigence system was used to monitor dynamic cell proliferation in real-time. Twenty-four hours after transfection with siRNA against DHRS7 or nontargeted control siRNA, LNCaP, BPH1, and PC3 cells were seeded in E-plates of the xCELLigene RTCA instrument and monitored for a further 48 h. Cell index refers to a relative change in electrical impedance representing the number of cells detected on the microelectrodes on the bottom of the plate. (B and C) Immunohistochemical staining of Ki-67 expression in LNCaP, BPH, and PC3 cells 48 h after knockdown of DHRS7, normalized to cells treated with control siRNA. Ki-67 index was determined by detecting the fraction of Ki-67 positively stained cells in five fields, scanned at 20× magnification using ImageJ. Representative images are shown. (D and E) Migration of LNCaP, BPH1, and PC3 cells after knockdown of DHRS7 assessed by the transwell migration assay. Cells transfected with siRNA against DHRS7 or scrambled nontargeted control siRNA were seeded on Boyden chamber transwell inserts at 24 h posttransfection, followed by crystal violet staining after another 24 h. Stained cells in five fields scanned at 10× magnification setting were analyzed using ImageJ. Representative pictures of Boyden chamber assays are shown. (F) Cell adhesion assay using fibronectin as an extracellular matrix in LNCaP, BPH1, and PC3 cells. At 24 h posttransfection with siRNA against DHRS7 or scrambled nontargeted control siRNA cells were seeded in fibronectin-coated plates. Adherent cells were quantified after 60 min by crystal violet staining. All data represent mean ± SD from at least three independent experiments conducted in triplicate. Statistical analysis was performed using the Student’s *t*-test; *P ≤ 0.0001 compared to the nontargeted control.

The impact of DHRS7 knockdown on cell proliferation was assessed using the xCELLigence system. Depletion of DHRS7 resulted in a threefold increase in LNCaP cell proliferation, which was supported by an enhanced Ki-67 staining (Fig.4A–C). The effect on cell proliferation upon knockdown of DHRS7 was verified by using an independent siRNA molecule (Fig. S2). In contrast to LNCaP, no significant changes in cell proliferation following DHRS7 depletion could be observed for PC3 and BPH1 cells, and also Ki-67 staining was not different between control and knockdown. Interestingly, depletion of DHRS7 had no effect on the cell cycle as detected by flow cytometric analysis (Fig. S4). These results suggest that knockdown of DHRS7 impairs cell proliferation, dependent on their basal proliferation rate and/or DHRS7 expression levels.

### Depletion of DHRS7 promotes cell migration and adhesion in PCa cells

To study the effects of DHRS7 downregulation on the migratory and adhesive capabilities of LNCaP, PC3, and BPH1 cells transwell cell migration and fibronectin adhesion assays were performed. Cell migration was significantly enhanced in cells treated with siRNA against DHRS7 compared with cells treated with nontargeted control siRNA (*P* < 0.05, Fig.4D and E). The effect was most pronounced in LNCaP cells where migration was increased threefold upon DHRS7 knockdown. The number of adherent cells following DHRS7 depletion was significantly reduced compared with controls (*P* < 0.05, Fig.4F). These results suggest that loss of DHRS7 promotes cell migration and decreases adhesion in all three cell lines tested.

### The impact of DHRS7 knockdown on the gene expression profile of LNCaP cells

**Figure 5:**
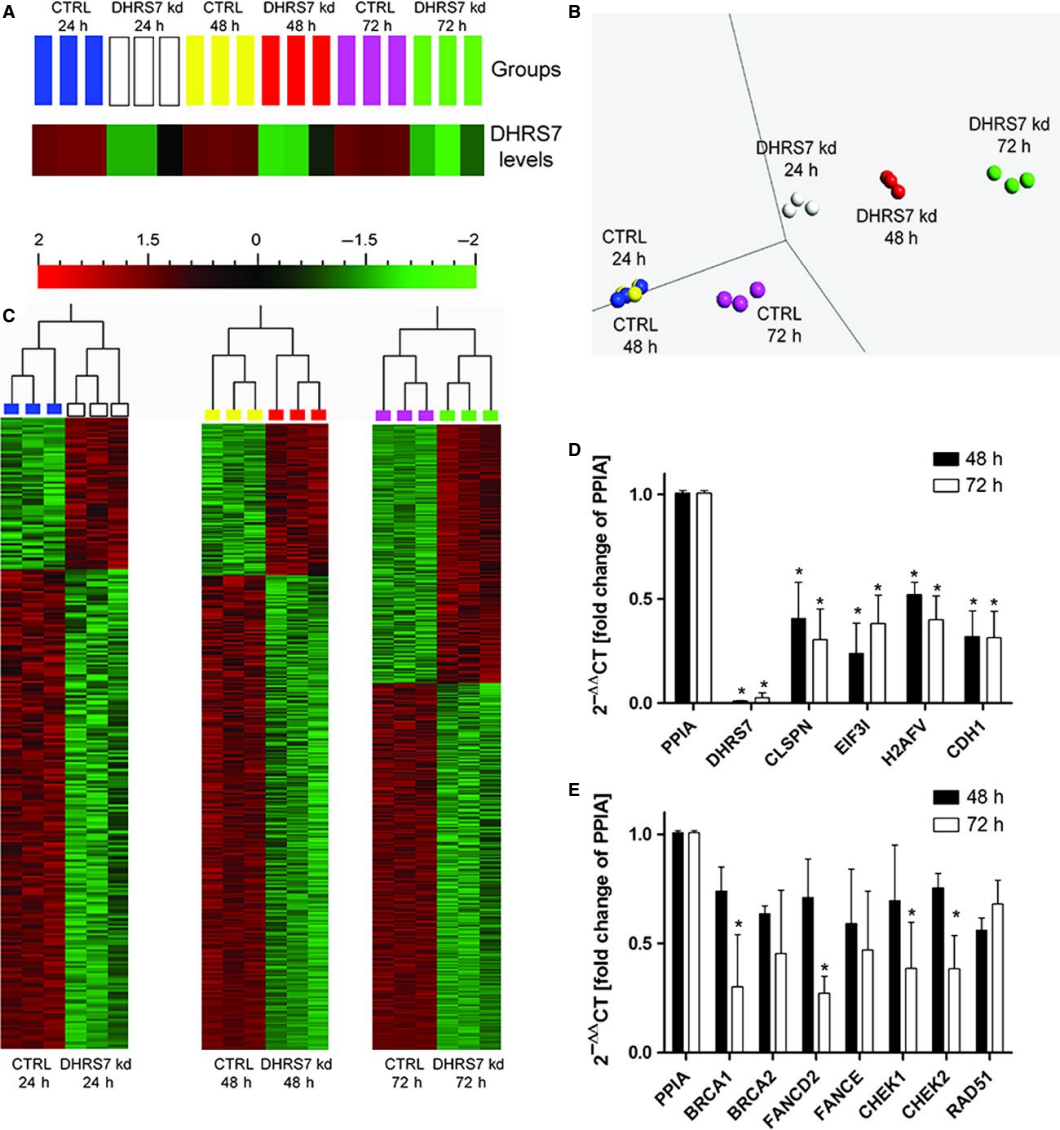
The impact of DHRS7 knockdown on the gene expression profile in LNCaP cells. (A) DHRS7 expression was efficiently decreased as early as 24 h after siRNA treatment. Color scheme representing normalized (−2 to 2) gene expression fold change. (B) PCA analysis showing profound alterations in the global gene expression profile of LNCaP cells upon DHRS7 knockdown. Each sphere represents one of the three replicates used for the microarray. Replicates samples obtained from DHRS7 knockdown cells cluster to each other but far away from the CTRL cells. Most profound gene expression profile differences in DHRS7 knockdown cells compared to CTRL are observed at 72 h after siRNA treatment. (C) Hierarchical clustering of samples based on significant differentially expressed genes (normalized fold-change −2.0 to 2.0) with (false discovery rate at *P* < 0.05) highlights major differences in gene expression among analyzed groups. (D) Validation of microarray data by qPCR on a selected pool of genes. (E) Genes involved in the BRCA1 pathway whose expression was altered in LNCaP cells upon DHRS7 knockdown. (D,E) Statistical analysis was performed using the Student’s t-test. *P < 0.05 compared to the nontargeted control.

On the basis of these in vitro findings, we investigated whether ablation of DHRS7 expression may impair the expression of genes involved in proliferation, migration, and adhesion. For this purpose we used LNCaP cells, due to their high-endogenous expression level and the pronounced effects of siRNA-mediated knockdown, and conducted a microarray study using the Affymetrix GeneChip Human Gene 2.0 ST Array. RNA was prepared at 24, 48, and 72 h posttransfection with siRNA against *DHRS7*. Nontargeting siRNA was used as control. First, we assured that *DHRS7* expression was efficiently decreased (Fig.5A). Following this, the transcriptome data were examined, revealing that DHRS7 knockdown altered the global gene expression profile of LNCaP cells as early as 24 h after siRNA treatment, as shown by principle component analysis (PCA) (Fig. 5A) analysis (Fig.5B) and hierarchical clustering (Fig.5C). The differences were more pronounced at 48 and 72 h (Fig.5B). To validate our microarray data, we performed qPCR to confirm some of the expression changes observed in the microarray following DHRS7 knockdown. The target genes were selected based on the fold change between control and DHRS7 knockdown treatment and due to their potential role in cell proliferation or metastasis, namely: *CLSPN*, *EIF3I*, *H2AFV*, and *CDH1* (Fig.5D). In order to determine whether the differentially expressed genes had functional relationships in similar signaling pathways, we employed the interactive pathway analysis (IPA) tool with Ingenuity software. IPA revealed enrichment of pathways involved in DNA replication, cellular growth and proliferation, cellular assembly and organization, migration, and adhesion as well as cancer (Fig. S5). Among the different pathways influenced by DHRS7 depletion, the BRCA1 pathway was one of the most affected. We also validated the expression of genes related to this pathway following DHRS7 knockdown by assessing the mRNA expression of *BRCA1*, *BRCA2*, *FANCD2*, *FANCE*, *CHEK1*, *CHEK2*, and *RAD51* by qPCR (Fig.5E). Together, these results suggest that DHRS7 knockdown alters the gene expression profile of LNCaP cells and supports our results described above concerning the effects on cell proliferation and migration.

## Discussion

Uncharacterized SDRs may play important physiological and pathological roles in multiple diseases, including cancer. Elucidation of their function is likely to provide an improved understanding of disease mechanisms, which is essential for the development of novel diagnostic and therapeutic applications 24. The “orphan” enzyme DHRS7 belongs to this enzyme family. Microarray-based gene expression profiling studies suggested that *DHRS7* expression is often decreased or even lost in PCa 9–11, raising the question about its potential role in tumor progression, however, its role in cancer has not been elucidated. Therefore, we decided to study the expression of DHRS7 in normal human prostate and in PCa tissue samples at different tumor stages. Furthermore, we assessed the effects of DHRS7 knockdown in vitro using different human prostate cell lines.

Through the analysis of hundreds of specimens from patients at different stages of disease, TMA technology has proven to serve as a powerful tool to promptly analyze clinical significance of new molecular markers in human tumors. Here, we took advantage of a prostate-specific TMA generated at our institution to asses DHRS7 expression in a large cohort of specimens (*n* = 348). Consistent with previously reported RNA-based microarray data 9–11, we verified that DHRS7 expression is diminished in PCa compared with normal prostate tissue samples on the protein level using TMA. Importantly, we report for the first time that DHRS7 protein levels are decreased in PCa tissues and negatively correlate with the GL. It remains to be established whether low-DHRS7 expression levels in primary PCa tissue may predict a high subsequent risk of distant metastases, as such finding would have significant potential for diagnostic and therapeutic implications. To date, we have not observed a clear correlation between DHRS7 expression levels and the survival of patients. However, this is not surprising in the context of PCa since it is in line with the lack of predictive value of important tumor suppressor genes. For example, although frequently mutated in 5–20% of PCa, the p53 status also failed to serve as a prognostic marker for survival in localized prostate adenocarcinoma while it serves as a useful marker in locally advanced PCa treated by androgen deprivation 25–27. It remains to be investigated whether changes in gene copy number variations (CNVs) are involved in the altered DHRS7 expression with increasing cancer state, since CNVs are generally observed with increasing tumor aggressiveness.

We then sought to support the protein expression-based findings from analysis of human tissues with a set of in vitro experiments using different prostate-derived cell lines. First, DHRS7 expression levels in LNCaP, PC3, and BPH1 cells were evaluated and then the effect of DHRS7 knockdown on key characteristics of aggressive cancer phenotypes like cell proliferation, migration and adhesion was investigated. DHRS7 knockdown led to a dramatically increased proliferation rate of LNCaP cells; however, no significant increase in cell proliferation was observed for PC3 and BPH1 cells. This may be explained by the fact that the basal proliferation rate of LNCaP cells is much lower (∼60 h doubling time) compared with that of PC3 and BPH1 cells (∼30–45 h doubling time) 28. Furthermore, LNCaP show very high DHRS7 expression, whereas PC3 and BPH1 express only moderate to low levels. It should be noted that the proliferation of LNCaP cells is androgen dependent, whereas that of PC3 and BPH1 cells has been shown to be androgen independent 29–31. Although the underlying mechanism remains unknown, it has been recently suggested that DHRS7 may promote de novo androgen synthesis, thus directly influencing the activation of the AR, thereby stimulating cancer progression 12. However, microarray analysis following DHRS7 knockdown in the present study did not show altered expression of genes involved in AR signaling, hence the verification of a possible role for DHRS7 in androgen-dependent signaling warrants further research.

Currently, the substrate(s) of DHRS7 remains unknown. Although a recent study suggested that DHRS7 might catalyze the reduction of several steroids, including 4-androstene-3,17-dione, as well as quinone containing xenobiotics, this evidence stems from indirect activity measurements and need to be confirmed. Using recombinant human DHRS7 expressed in HEK-293 cells, we were unable to detect any activity on cortisone/cortisol, estrone/estradiol and 4-androstene-3,17-dione/testosterone, in contrast to the potent activities that we observed for 11*β*-HSD1, 17*β*-HSD1, and 17*β*-HSD3 using these substrate/product pairs (data not shown). It remains to be investigated whether DHRS7 might play a role in the production of androgens via the backdoor pathway 32 or whether it indirectly stimulates androgen-dependent cancer cell proliferation.

To evaluate a possible influence of DHRS7 on cell cycle, LNCaP cells were analyzed by flow cytometry. Nevertheless, no significant changes could be detected, (Fig. S4), indicating that DHRS7 knockdown is increasing proliferation of LNCaP cells without affecting the cell cycle. In addition to the changes in LNCaP cell proliferation, increased cell migration as well as decreased cell adhesion was observed for all three cell lines tested. These results are further supported by our microarray data analysis that underlines the effect of DHRS7 depletion on the expression of genes involved in cell proliferation and cell adhesion pathways in LNCaP cells. Moreover, the transcriptome profiling of DHRS7 knockdown versus control LNCaP cells revealed a decreased expression of *CDH1* (also known as E-cadherin). Loss of E-cadherin promotes the transition of epithelial cells to the mesenchymal state (EMT), which is observed in metastasis 33. E-cadherin is an important switch in EMT, which could explain the increased migration and adhesion observed in the prostate cell lines. Another possible mechanism for the DHRS7-mediated regulation of PCa progression could involve the BRCA1/2 pathway that was also affected based on the data from our microarray analysis. BRCA1 and BRCA2 both are prostate tumor suppressors and their loss is associated with enhanced cell proliferation and overall cancer progression 34,35. Nevertheless, further research is needed to investigate the impact of DHRS7 function on these pathways, since these effects were only detected 48 and 72 h following knockdown, but not after 24 h, and may therefore represent secondary effects.

In conclusion, our in vitro experiments provide compelling evidence for DHRS7 as a key regulator of PCa cancer cell properties. According to our results and those of others, DHRS7 possesses tumor suppressor functions in PCa. Nevertheless, the mechanism underlying the effects of DHRS7 on cancer cell (and normal cell) behavior remains unknown and warrants further research. Future studies are required to identify the substrate(s) and product(s) of DHRS7 and to elucidate its regulation of expression. A better understanding of the tumor-suppressive role of DHRS7 may lead to the identification of a novel therapeutic PCa target and/or the potential development of a diagnostic application.

## Supporting Information

**Figure S1.** Comparison of the knockdown efficiency of four different siRNAs against DHRS7 mRNA levels. Four siRNAs against DHRS7 were assessed by single application and as pooled siRNA mixture at a concentration of 15 nmol/L for gene silencing in LNCaP cells after 24, 48, and 72 h. As a control, nontargeted siRNA was used. DHRS7 mRNA expression was normalized to the housekeeping gene for cyclophilin A (PPIA).

**Figure S2.** Comparison of cell proliferation in LNCaP cells after knockdown of DHRS7 with two different siRNAs. The xCELLigence system was used to monitor dynamic cell proliferation in real-time. Following 24 h of transfection with siRNA no. 2 or no. 4 against DHRS7 or nontargeted control siRNA, respectively, LNCaP cells were seeded in E-plates of the xCELLigene RTCA instrument and monitored for a further 48 h.

**Figure S3.** DHRS7 representative staining in human normal prostate and PCa samples. The spectrum of DHRS7 staining intensity ranges from absent/low (0) to very high/strong (3), from left to right side, respectively, in tested samples. Normal prostate to PCa GL5 specimens are reported, going from up to down side, respectively.

**Figure S4.** Histogram showing the percentages of cells in each phase of the cell cycle after DHRS7 knockdown. LNCaP cells were treated with siRNA against DHRS7 and collected after 24 or 48 h. Subsequently, cells were subjected to propidium iodide staining and analyzed for DNA content by flow cytometry.

**Figure S5.** Interactive pathway analysis (IPA)-based gene enrichment analysis. Graphical representation of most alerted diseases and cell functions (A), toxicity function related pathways (B), and cell cycle associated genes (C) in DHRS7 knockdown cells.

**Table S1.** Human oligonucleotide primers used for qPCR.
