# Supplementary figures and images for: A role for the dehydrogenase DHRS7 (SDR34C1) in prostate cancer
Source: Cancer Med. 2015 Aug 26;4(11):1717–29. doi: 10.1002/cam4.517 (PMC4673999; doi:10.1002/cam4.517)

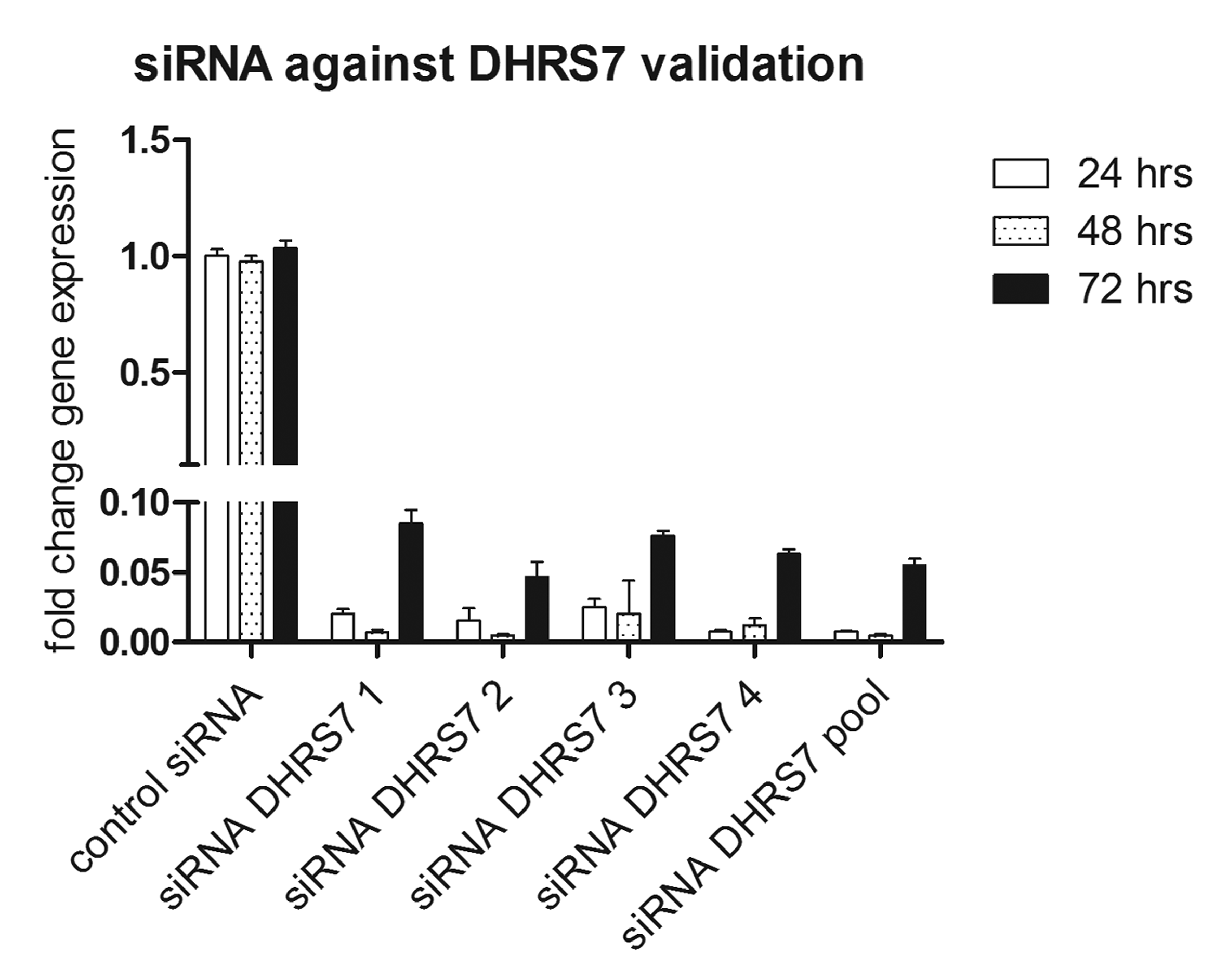

Supplement: Supplementary file 1 [file cam40004-1717-sd1.tif]

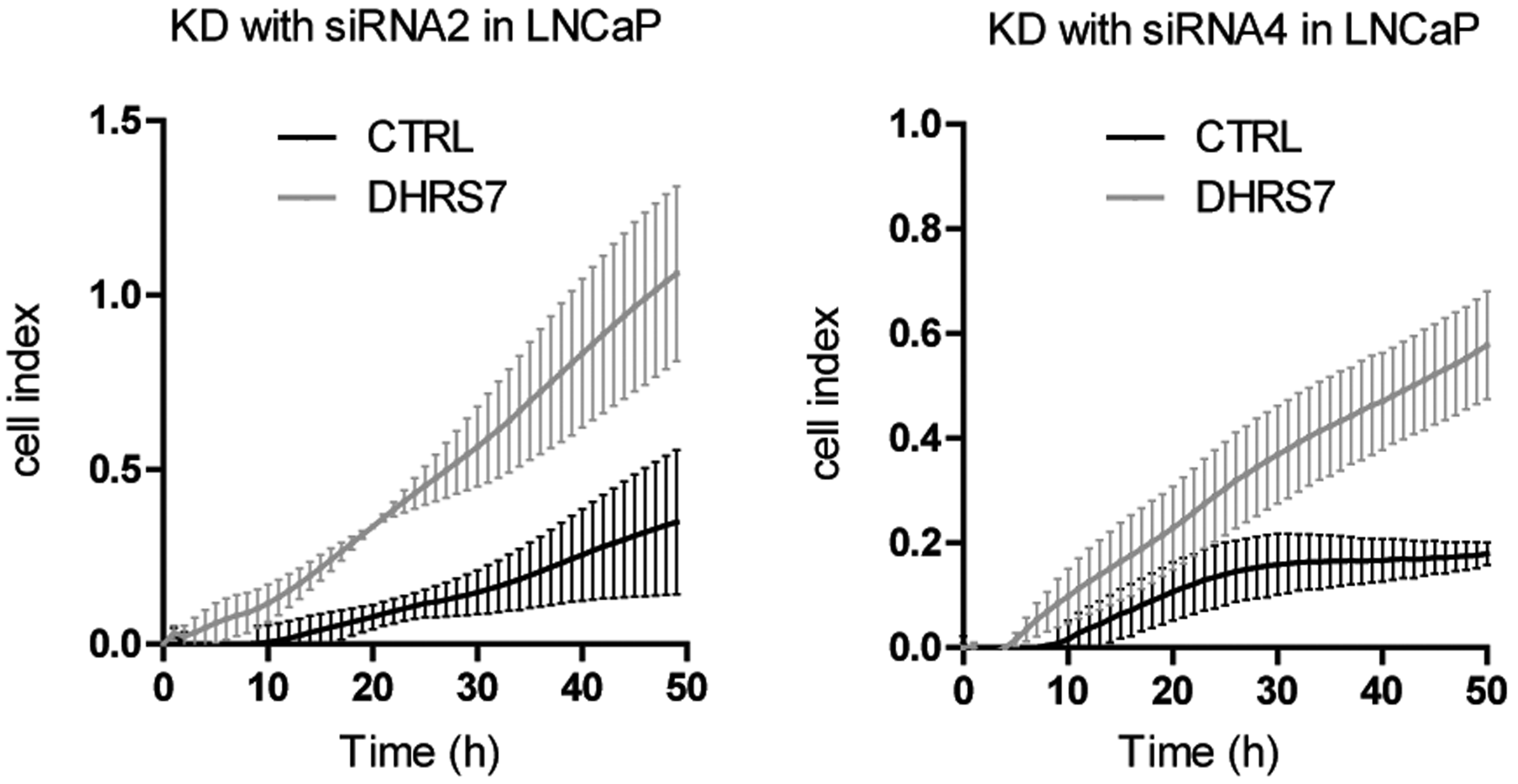

Supplement: Supplementary file 2 [file cam40004-1717-sd2.tif]

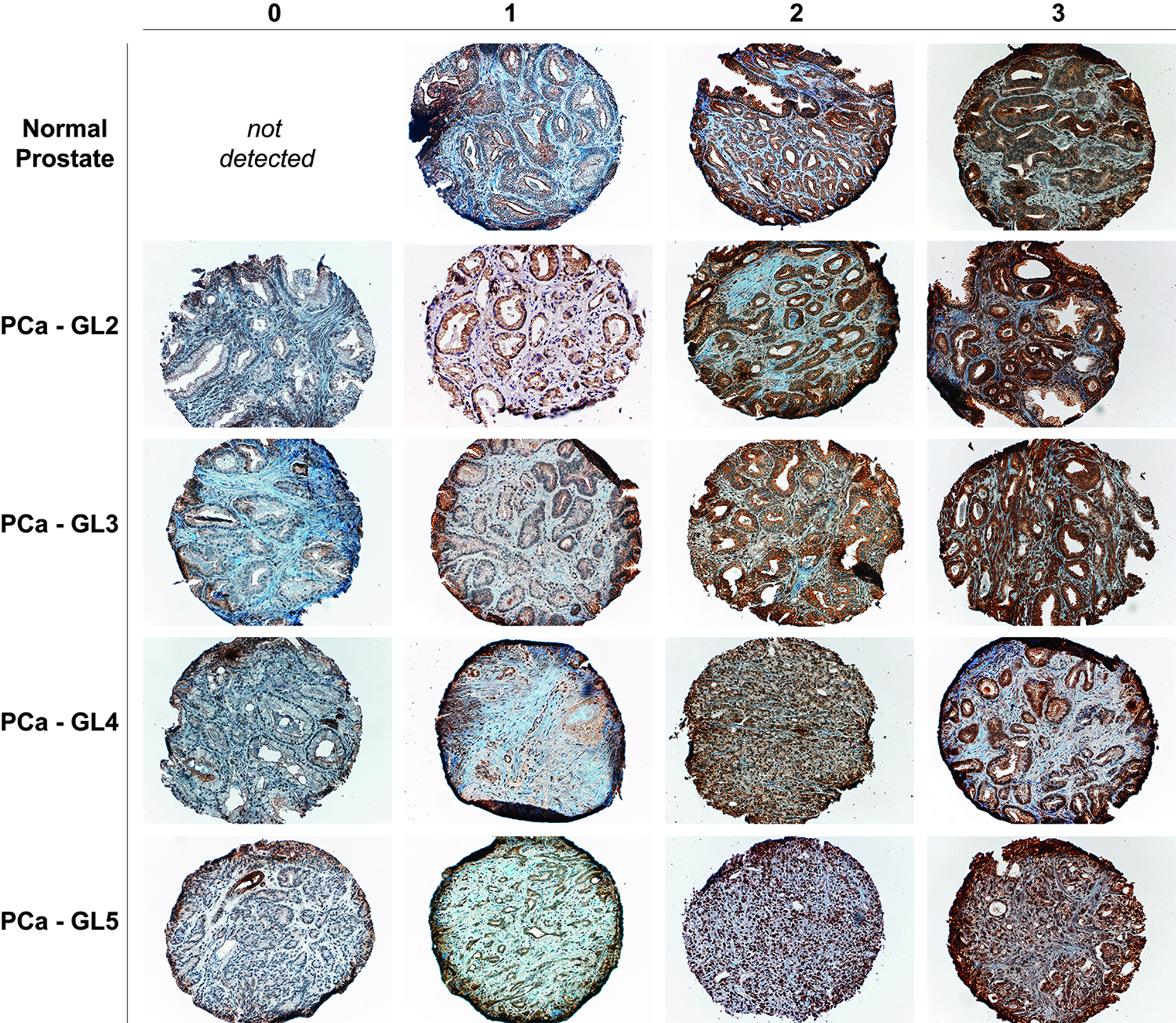

Supplement: Supplementary file 3 [file cam40004-1717-sd3.tif]

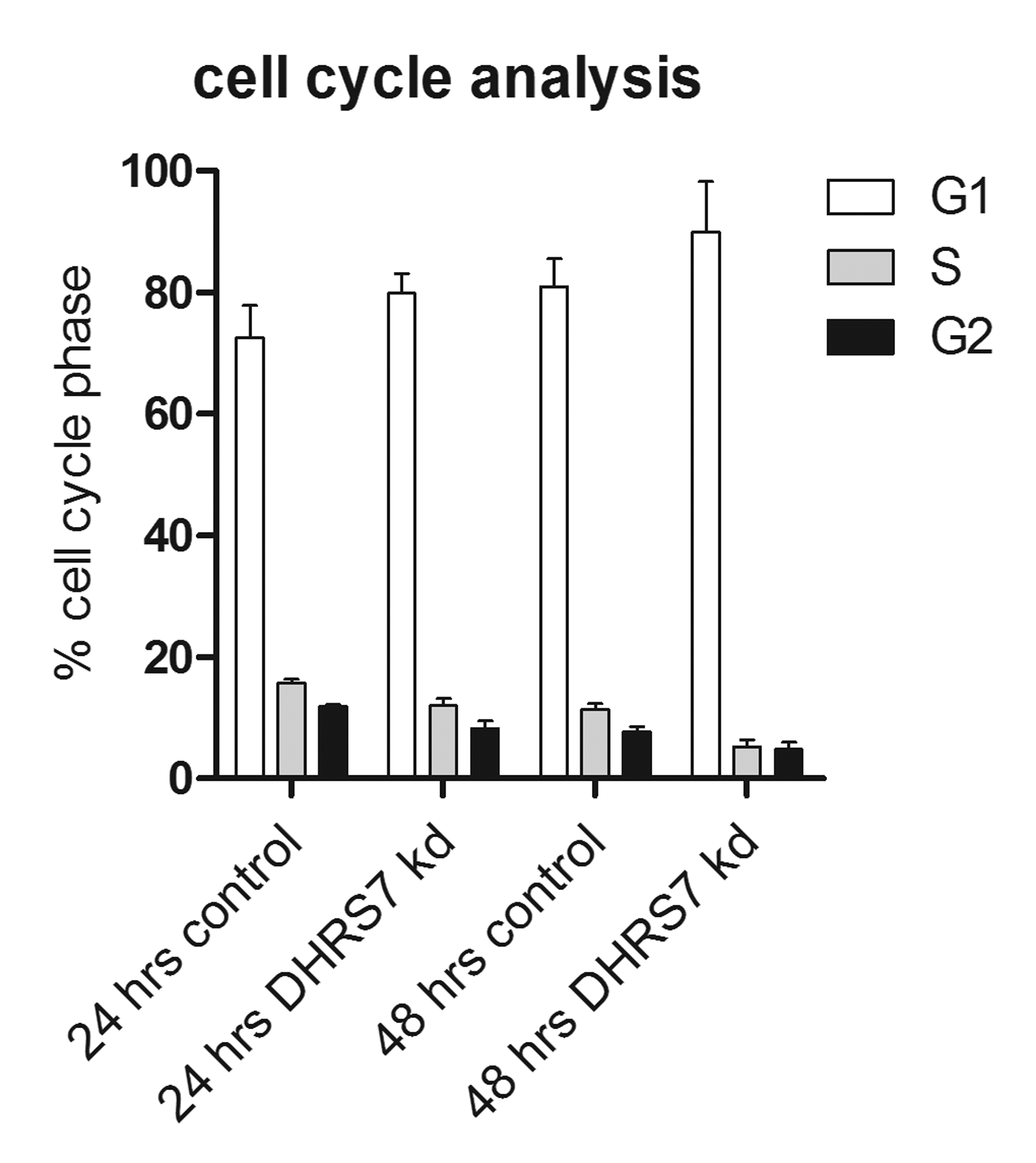

Supplement: Supplementary file 4 [file cam40004-1717-sd4.tif]

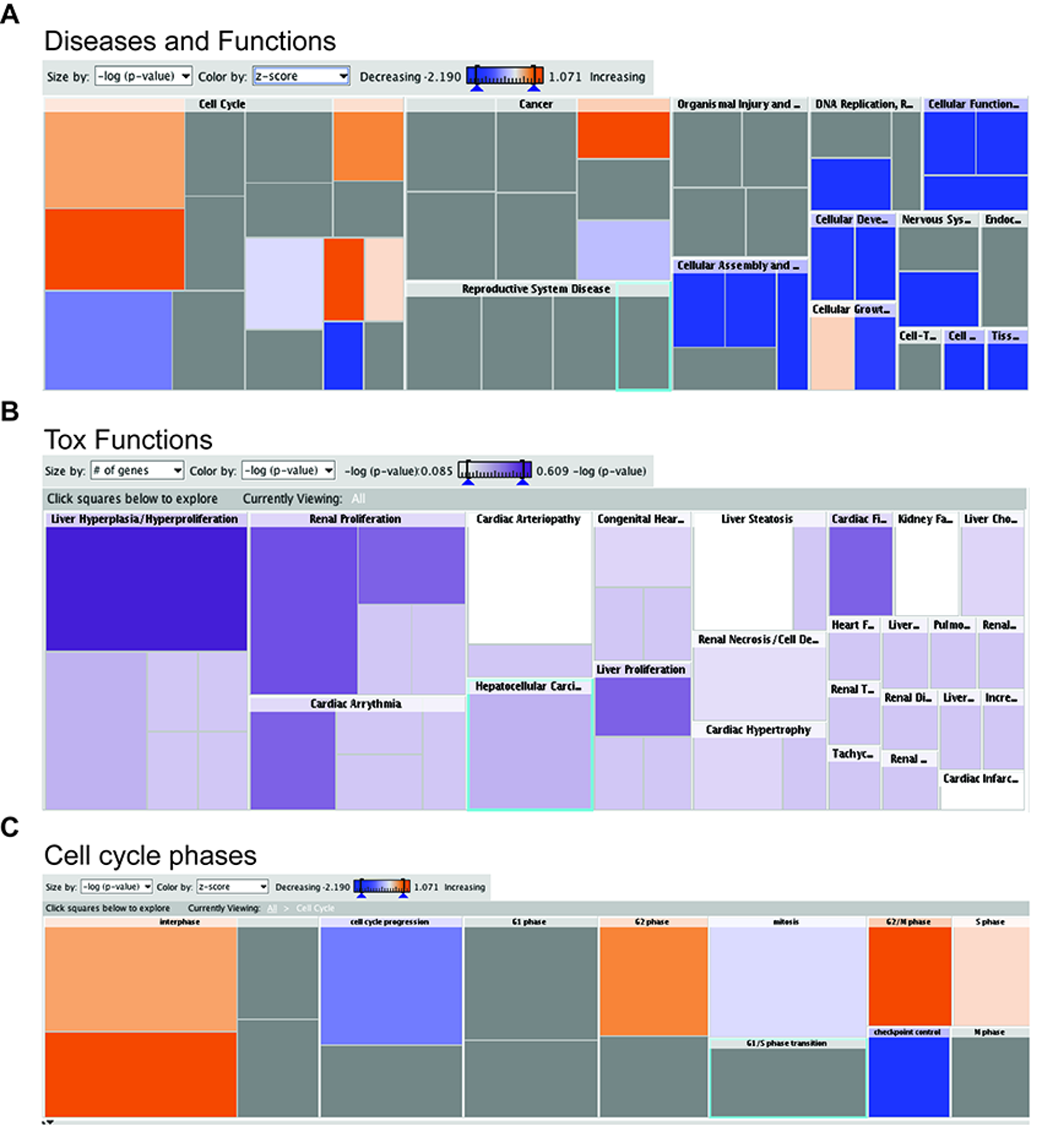

Supplement: Supplementary file 5 [file cam40004-1717-sd5.tif]
