# Supplementary material for: A role for the dehydrogenase DHRS7 (SDR34C1) in prostate cancer
Source: Cancer Med. 2015 Aug 26;4(11):1717–29. doi: 10.1002/cam4.517 (PMC4673999; doi:10.1002/cam4.517)
Supplement: Supplementary file 6 [file cam40004-1717-sd6.doc]

Supplementary Information

**A role for the dehydrogenase DHRS7 (SDR34C1) in prostate cancer**

Julia Seibert^a^, Luca Quagliata^b^, Cristina Quintavalle^b^, Thomas G. Hammond^a^, Luigi Terracciano^b^, Alex Odermatt^a^

**Methods**

Cell cycle assay

Following incubation with untargeted siRNA or siRNA for knockdown of DHRS7 for 24 h and 48 h, the LNCaP cells were harvested and fixed overnight in 70% ethanol at 4°C. The fixed cells were washed with PBS, then stained with propidium iodide (50 μg/ml) in the presence of RNAse A (50 μg/ml ) for 1 h at room temperature in the dark. The stained cells were assessed using flow cytometry (FACS Canto II flow cytometer, Becton Dickinson) and analyzed by FlowJo VX software (TreeStar).

**Supplementary Figure Legends**

**Supplementary Figure 1:** **Comparison of the knockdown efficiency of four different siRNAs against DHRS7 mRNA levels**. Four siRNAs against DHRS7 were assessed by single application and as pooled siRNA mixture at a concentration of 15 nM for gene silencing in LNCaP cells after 24 h, 48 h and 72 h. As a control, non-targeted siRNA was used. DHRS7 mRNA expression was normalized to the housekeeping gene for cyclophillin A (PPIA).

**Supplementary Figure 2:** Comparison of cell proliferation in LNCaP cells after knockdown of DHRS7 with two different siRNAs. The xCELLigence system was used to monitor dynamic cell proliferation in real-time. Following 24 h of transfection with siRNA No.2 or No.4 against DHRS7 or non-targeted control siRNA, respectively, LNCaP cells were seeded in E-plates of the xCELLigene RTCA instrument and monitored for a further 48 h.

**Supplementary Figure 3:** DHRS7 representative staining in human normal prostate and PCa samples. The spectrum of DHRS7 staining intensity ranges from absent/low (0) to very high/strong (3), from left to right side respectively, in tested samples. Normal prostate to PCa GL5 specimens are reported, going from up to down side respectively.

**Supplementary Figure 4:** Histogram showing the percentages of cells in each phase of the cell cycle after DHRS7 knockdown. LNCaP cells were treated with siRNA against DHRS7 and collected after 24 h or 48 h. Subsequently, cells were subjected to propidium iodide staining and analyzed for DNA content by flow cytometry.

**Supplementary Figure 5:** Interactive pathway analysis (IPA)-based gene enrichment analysis. Graphical representation of most alerted diseases and cell functions (A), toxicity function related pathways (B) and cell cycle associated genes (C) in DHRS7 knockdown cells.

**Supplementary Table 1.**

**Human oligonucleotide primers used for qPCR**

| **Gene** | **Primer** | |
| --- | --- | --- |
|  | **forward (5'-3')** | **reverse (5'-3')** |
| **BRCA1** | GAAACCGTGCCAAAAGACTTC | CCAAGGTTAGAGAGTTGGACAC |
| **BRCA2** | CACCCACCCTTAGTTCTACTGT | CCAATGTGGTCTTTGCAGCTAT |
| **CDH1** | CGAGAGACTACACGTTCACGG | GGGTGTCGAGGGGAAAAATAGG |
| **CHEK1** | ATATGAAGCGTGCCGTAGACT | TGCCTATGTCTGGCTCTATTCTG |
| **CHEK2** | TCTCGGGACTCGGATGTTGAG | CCTGAGTGGACACTGTCTCTAA |
| **DHRS7** | GAGTTTGGTAGAATCGACTTTCTG | GAAAGAGGTACAGATATGATACCC |
| **FANCD2** | AAAACGGGAGAGAGTCAGAATCA | ACGCTCACAAGACAAAAGGCA |
| **PPIA** | CATCTGCACTGCCAAGACTGA | TGCAATCCAGCTAGGCATG |
| **RAD51** | CAACCCATTTCACGGTTAGAGC | TTCTTTGGCGCATAGGCAACA |
